# Complete Genome Sequence of the Linear Plasmid pJD12 Hosted by *Micrococcus* sp. D12, Isolated from a High-Altitude Volcanic Lake in Argentina

**DOI:** 10.1128/genomeA.00627-15

**Published:** 2015-06-11

**Authors:** Julian Rafael Dib, Angel Angelov, Wolfgang Liebl, Johannes Döbber, Sonja Voget, Jörg Schuldes, Marta Gorriti, Maria Eugenia Farías, Friedhelm Meinhardt, Rolf Daniel

**Affiliations:** a PROIMI-CONICET, Tucumán, Argentina; b Department of Microbiology, Universidad Nacional de Tucumán, Tucumán, Argentina; c Lehrstuhl für Mikrobiologie, Technische Universität München, Freising, Germany; d Institut für Molekulare Mikrobiologie und Biotechnologie, Westfälische Wilhelms-Universität, Münster, Germany; e Genomic and Applied Microbiology and Göttingen Genomics Laboratory, Georg-August University, Göttingen, Germany

## Abstract

The linear plasmid pDJ12 from *Micrococcus* D12, isolated from the high-altitude volcanic Diamante Lake in the northwest of Argentina, was completely sequenced and annotated. It is noteworthy that the element is probably conjugative and harbors genes potentially instrumental in coping with stress conditions that prevail in such an extreme environment.

## GENOME ANNOUNCEMENT

Micrococci are widely distributed and have been isolated from diverse locations including extreme sites (1–4). Among members of the phylum *Actinobacteria*, the genus *Micrococcus* is of increasing biotechnological importance; *Micrococcus* species can be used for biodegradation and bioremediation processes (5, 6), and they can be applied for producing useful compounds such as industrially relevant enzymes (7, 8) and long-chained alkenes for the environmentally friendly substitution of fossil fuels (9). *Micrococcus* sp. D12 was previously found to host the large approximately 75-kb plasmid pJD12. Further characterization revealed that the plasmid is a linear rather than a circular extrachromosomal genetic element (3).

Here, we present the complete genome sequence of the linear plasmid pJD12 hosted by *Micrococcus* sp. D12. The plasmid pJD12 was isolated by pulse field gel electrophoresis and sequenced by a combination of Sanger and 454 pyrosequencing. A plasmid library was constructed applying the TOPO TA kit as recommended by the manufacturer (Life Technologies, Darmstadt, Germany). In total, 192 recombinant plasmids were end sequenced with an ABI 3730xl automated DNA sequencer using BigDye chemistry (Life Technologies, Darmstadt, Germany). Obtained sequences were processed with Phred and assembled using Phrap (http://www.phrap.org). The 454 shotgun library was generated and sequenced with the Genome Sequencer FLX system using titanium chemistry as recommended by the manufacturer (454 Life Sciences, Roche Applied Science, Branford, USA). Approximately 10,000 shotgun reads were generated and assembled *de novo* using the Roche Newbler assembler software v2.9 (454 Life Sciences, Roche Applied Science). Subsequently, contigs generated by the Sanger-sequencing and the pyrosequencing were joined, yielding three large contigs. Remaining gaps were closed by PCR and Sanger sequencing. Finally, the lacking terminal sequences were determined following a self-ligation method (10). The plasmid’s termini were obtained by restriction with AfeI, which cuts close the telomeric regions, followed by self-ligation and PCR amplification of the unknown DNA and Sanger sequencing. Annotation was performed by the Integrated Microbial Genomes (IMG) annotation pipeline (11).

The plasmid pJD12 consists of linear DNA spanning 75,989 bp with an average G+C content of 68.8%. The annotation revealed the presence of 80 putative open reading frames (ORFs). The element encodes plasmid typical genes, including those for conjugation and replication. Interestingly, it also contains genetic information encoding a glutaredoxin and a putative cobalt-zinc-cadmium efflux system, which are potentially involved in coping with oxidative stress and heavy-metal poisoning, and may be favorable for the host survival and growth in the hostile environment. Moreover, because the lack of efficient genetic tools for Micrococci, pJD12 as a potential conjugative element may serve as the basis of novel vectors for genetic engineering.

### Nucleotide sequence accession numbers.

The genome sequence of linear plasmid pJD12 has been deposited in the GenBank database under the accession no. KR152226. The version described here is version KR152226.1.
